# A Multidimensional Bibliometric Analysis of Research Hotspots and Development Trends in Functional Microorganisms of the Genus *Geobacter*

**DOI:** 10.3390/microorganisms14081678

**Published:** 2026-07-31

**Authors:** Lisi Jiang, Jianing Zhang, Chenxu Wang, Junying Wu, Wenyuan Li, Jiajun Fan, Lixin Guo, Chang Guo, Yang Zhang

**Affiliations:** College of Life Science, Shenyang Normal University, Shenyang 110034, China; jianglisi@synu.edu.cn (L.J.); zhangjn0421@163.com (J.Z.); wang333308@163.com (C.W.); wujunying1126@126.com (J.W.); lixiaohan20040106@163.com (W.L.); fanjiajun369@126.com (J.F.); 15840469607@163.com (L.G.); 13942908475@163.com (C.G.)

**Keywords:** bibliometrics, *Geobacter*, extracellular electron transfer (EET), microbial fuel cells (MFCs), biogeochemical cycles, bioremediation

## Abstract

Rapid industrialization, urbanization, resource exploitation, and energy use have caused global pollution and ecological degradation, hindering sustainable development. Functionally versatile microbes are eco-friendly resources for addressing environmental problems. *Geobacter*, a vital genus featuring outstanding extracellular electron transfer (EET), has drawn widespread research interest, with studies centered on EET mechanisms, microbial fuel cells, biogeochemical cycling and environmental bioremediation. Though valuable across energy, environmental and geoscience disciplines, a systematic bibliometric review of *Geobacter* research evolution is absent. This paper fills the gap via comprehensive bibliometric analysis of 2000–2026 Web of Science Core Collection papers covering *Geobacter* and the closely related genus *Trichlorobacter*, recently re-established as a standalone genus, focusing on bioremediation, dissimilatory Fe(III) reduction, microbial electrochemistry and biogeochemical cycles. This bibliometric profiling clarifies the evolving research frontiers in electroactive microbe-mediated environmental remediation and geochemical cycling, delivering refined scientific implications and actionable theoretical guidance for future ecological restoration and pollution remediation practices.

## 1. Introduction

With the continuous aggravation of global environmental pollution and ecosystem degradation, developing efficient, sustainable, and eco-friendly technologies for pollution mitigation and resource recovery has become a core research focus in modern environmental science [1]. Functional microorganisms, characterized by diverse metabolic functions and strong environmental adaptability, have emerged as crucial biological tools to address global ecological and environmental crises [2,3]. Among these, the genus *Geobacter* displays distinctive physiological traits and research emphases. *Geobacter* mediates extracellular electron transfer (EET) primarily through conductive pili and outer-membrane c-type cytochromes, enabling highly efficient electron transfer without the need for exogenous electron mediators. This mechanism clearly distinguishes it from facultative anaerobes such as *Shewanella*, which relies predominantly on flavin-based electron shuttling, and *Pseudomonas*, which employs phenazine mediators for electron transfer and exhibits a broad metabolic substrate range. Current research on *Geobacter* is closely aligned with fundamental scientific questions concerning subsurface biogeochemical cycling and in situ anaerobic bioremediation of heavy metals and recalcitrant organic pollutants. Findings from this work directly inform the development of bioremediation strategies tailored to natural anaerobic environments—including groundwater systems and sediments. In contrast, studies on *Shewanella* and *Pseudomonas* predominantly focus on wastewater treatment under aerobic or facultative anaerobic conditions, as well as on the engineering applications of bioelectrochemical systems. Consequently, *Geobacter* serves as the central model organism for elucidating microbially driven natural element cycling and for advancing bioremediation technologies targeting anaerobic habitats.

*Geobacter* belongs to the class Deltaproteobacteria, phylum Proteobacteria (distinct from Actinobacteria), and is defined as a genus of Gram-negative, obligately anaerobic, non-motile bacteria without flagella [4]. Widely distributed in natural and engineered environments, including marine and freshwater sediments, soil ecosystems, river systems, and wastewater treatment facilities, *Geobacter* is recognized as a typical obligate iron-reducing bacterium. Its remarkable metabolic versatility enables active participation in key biogeochemical processes, particularly the cycling of carbon, nitrogen, and iron elements [5,6,7], which profoundly drives material migration and elemental homeostasis in terrestrial and aquatic ecosystems. Furthermore, *Geobacter* can efficiently degrade refractory aromatic hydrocarbons such as benzene and toluene, and remove heavy metal contaminants (e.g., Cr(VI), U(VI)) via biosorption and enzymatic reduction [8,9], providing reliable biological strategies for the remediation of complex contaminated sites. Notably, the highly efficient EET capacity of *Geobacter*, mediated by conductive pili (i.e., microbial nanowires) and outer membrane cytochromes, lays a critical mechanistic foundation for its application in microbial fuel cells (MFCs), bioelectrochemical systems (BESs), and other energy conversion technologies [10,11], offering a core microbial platform for sustainable bioenergy development. Given the expanding application prospects of *Geobacter* in environmental biotechnology, pollution remediation, and bioenergy engineering, it is imperative to systematically explore its research evolution, identify emerging frontiers, and predict future development trends, so as to advance both basic theoretical research and practical translational applications.

Previous studies have accumulated extensive findings on the elucidation of *Geobacter* physiological mechanisms and the development of applied technologies. Relevant reviews have also systematically summarized the enhancement effects of metabolic engineering and interspecies interactions of *Geobacter sulfurreducens* on microbial fuel cell (MFC) performance, and identified publication trends and research hotspots in the MFC field through bibliometric analysis.

Overall, however, there remains a lack of comprehensive panoramic bibliometric analysis centered on the entire *Geobacter* genus and covering four core research directions: fundamental mechanisms of extracellular electron transfer (EET), MFC applications, biogeochemical cycle functions, and pollution bioremediation practices [12]. Independent studies focusing on a single application direction fail to systematically reveal the overall evolutionary trajectory of the field over the past two decades and the logical knowledge connections across different research directions. Furthermore, there is still a dearth of systematic quantitative elucidation of meso-level scientific questions, including the global distribution of research capacity, characteristics of institutional collaboration networks, and evolutionary paths of thematic hotspots.

In this context, the present study adopts a bibliometric approach based on the Web of Science (WoS) Core Collection database. All relevant peer-reviewed publications published from 2000 to 2026 focusing on *Geobacter* and the taxonomically revised genus *Trichlorobacter* were systematically retrieved and screened, with research scopes covering bioremediation, dissimilatory iron reduction, microbial fuel cells, and biogeochemical cycling. Based on the collected literature dataset, comprehensive quantitative and visual analyses were performed using CiteSpace 6.3.R1 and VOSviewer 1.6.20 software. Multiple research dimensions were explored, including annual publication trends, national and institutional collaboration networks, core authors, high-impact journals, keyword co-occurrence and thematic clustering, and document co-citation characteristics. This study aims to: (i) systematically map the knowledge structure and evolutionary trajectory of global *Geobacter* research over the past two decades; (ii) identify mainstream research hotspots and existing knowledge gaps; (iii) forecast emerging research trends and future developmental directions; (iv) provide solid empirical evidence and theoretical support for subsequent basic research and translational applications; and (v) promote the innovative application of *Geobacter*-based biotechnology in ecological restoration and sustainable energy development, thereby offering feasible biological solutions for global ecological environmental governance.

## 2. Materials and Methods

### 2.1. Data Sources and Retrieval

This study was prepared following the Preferred Reporting Items for Systematic Reviews and Meta-Analyses (PRISMA) statement (Supplementary Table S1) [13], and its research protocol was retrospectively pre-registered on the Open Science Framework Registries with the identifier: https://doi.org/10.17605/OSF.IO/3STGE.

**Figure 1:**
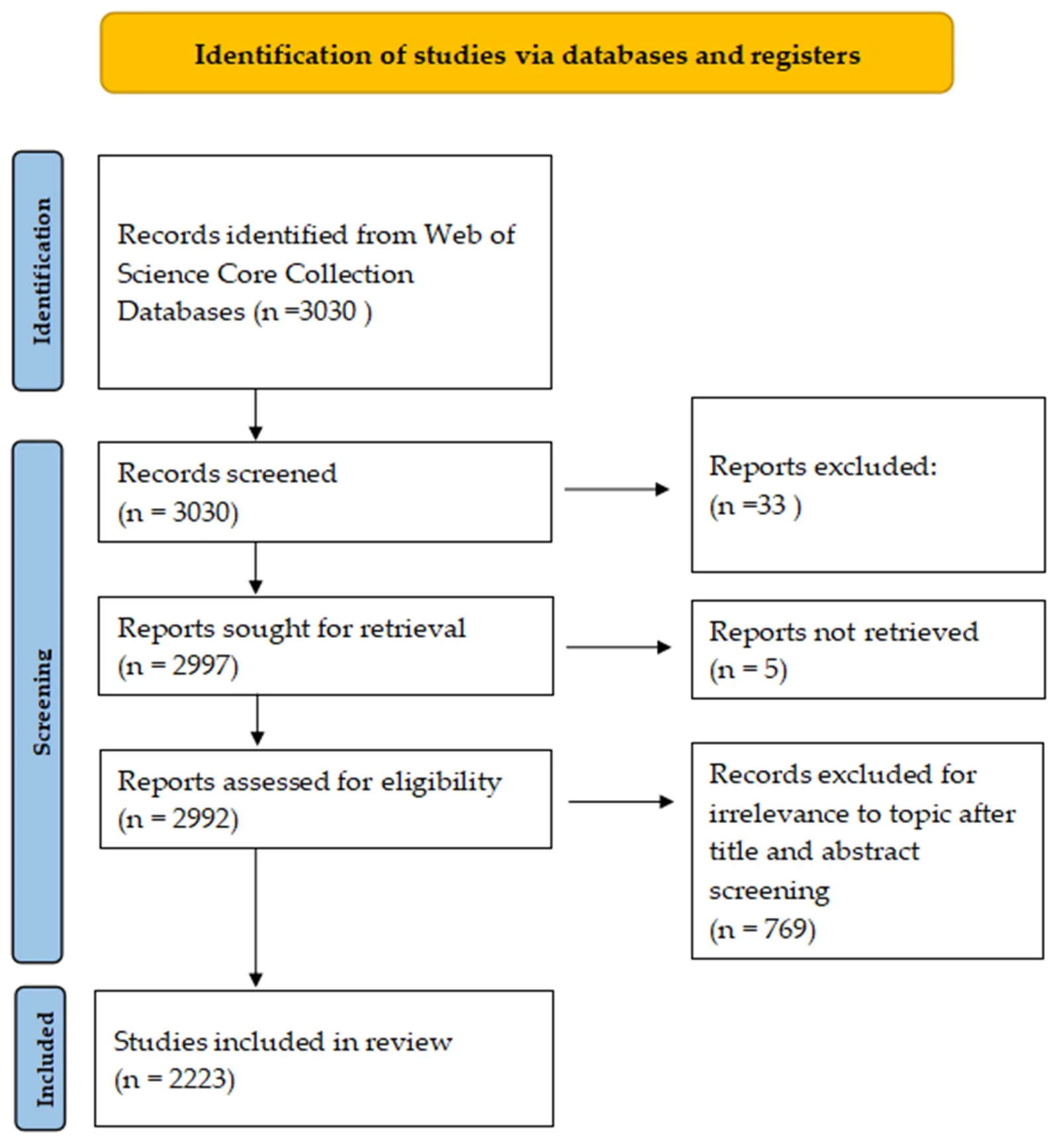
Screening flowchart of comprehensive data screening procedure.

In this context, this study performed a systematic bibliometric analysis based on the literature data retrieved from the Web of Science (WoS) Core Collection. As an authoritative and multidisciplinary bibliographic database, WoS provides high-quality, peer-reviewed academic resources and was widely recognized as the most reliable data source for global bibliometric research, with established academic credibility and authority [14]. To guarantee temporal uniformity and eliminate statistical bias from continuously updated online publications, all literature retrieval procedures were finalized at 22:58 on 25 January 2026. Retrievals were restricted to the Science Citation Index Expanded (SCI-EXPANDED) and Social Sciences Citation Index (SSCI) databases, covering publications issued between 2000 and 2026. Only original articles and review articles were retained for subsequent analysis. Literature retrieval was implemented via the WoS QUERY BUILDER interface with the following search formula: TS = ((“*Geobacter*”) OR (“*Trichlorobacter*”)) AND TS = (((“bioremediation” OR “environmental remediation” OR “soil remediation” OR “water treatment”) OR (“iron reduction”) OR (“organic degradation”) OR (“organohalide-respiring”)) OR ((“electroactive bacteria” OR “electrogenic bacteria”) OR (“microbial fuel cell” OR “MFC” OR “bioelectrochemical system” OR “BES” OR “microbial electrolysis cell”) OR (“extracellular electron transfer” OR “EET” OR “nanowire” OR “pilus” OR “c-type cytochrome”)) OR ((“biogeochemical cycle” OR “nitrogen cycle” OR “iron cycle”) OR (“syntrophy” OR “microbial community” OR “interspecies electron transfer”))). Initially, a total of 3030 publications were retrieved based on the established search criteria. All judgment criteria, including inclusion criteria, exclusion criteria, and definition of literature types, were jointly formulated by the authors. Two groups of two researchers each conducted independent literature screening. Disputed articles on which screeners failed to reach a consensus were submitted to arbitrators for final adjudication. A total of 2223 valid studies were retained after screening, and the screening workflow is shown in Figure 1. All qualified studies were exported in the format of “Full Record and Cited References” to support subsequent quantitative bibliometric analysis and in-depth content mining.

### 2.2. Statistical Methods

Bibliometrics has emerged as a robust quantitative discipline for systematic domain analysis. Originating from the pioneering statistical literature research reported by Cole and Eales in 1917, this field underwent continuous theoretical and methodological refinement over decades. In 1969, Pritchard formally coined the term “bibliometrics” to replace “statistical bibliography”, marking the disciplinary maturity and standardization of bibliometric research [15]. As a well-established analytical framework, bibliometrics integrates statistical quantification and visual mining to decode core bibliographic indicators, including authors, keywords, journals, countries, institutions, and citations. This approach enables the comprehensive elucidation of the knowledge evolution, internal structural features, and emerging research frontiers of a specific academic domain.

CiteSpace 6.3.R1, a classic scientometric tool developed by the research team at Drexel University, USA, was utilized for knowledge network analysis in this study. Supported by rigorous scientometric theories and visual computational algorithms, this software facilitates high-precision knowledge mapping based on citation relationships. Its unique Timezone and Timeline functions can dynamically track the temporal evolution of research themes and the clustering characteristics of hot topics over time [16]. In this study, CiteSpace was applied to construct multiple analytical maps for *Geobacter* research, including annual publication distribution, dual-map overlay, co-citation network, keyword co-occurrence, and keyword burst detection.

VOSviewer, an open-access bibliometric software developed by the Center for Science and Technology Studies of Leiden University (Netherlands), is widely adopted for its streamlined operation and high-quality visualized outputs. It supports multi-dimensional co-occurrence analysis and generates standardized network, overlay, and density maps for bibliometric datasets [17]. Herein, VOSviewer was employed to investigate author collaboration patterns, document co-occurrence associations, and co-citation networks, aiming to systematically reveal the collaborative architecture and knowledge relevance of global *Geobacter* research.

Tableau, a versatile data visualization platform with strong multi-source data compatibility, was adopted in this study for spatial statistical analysis. The platform was used to visualize the global geographical distribution of retrieved publications, which intuitively reflected the spatial variation in research output and the academic contributions of different countries and regions in the field of *Geobacter* research [18].

## 3. Results

**Figure 2:**
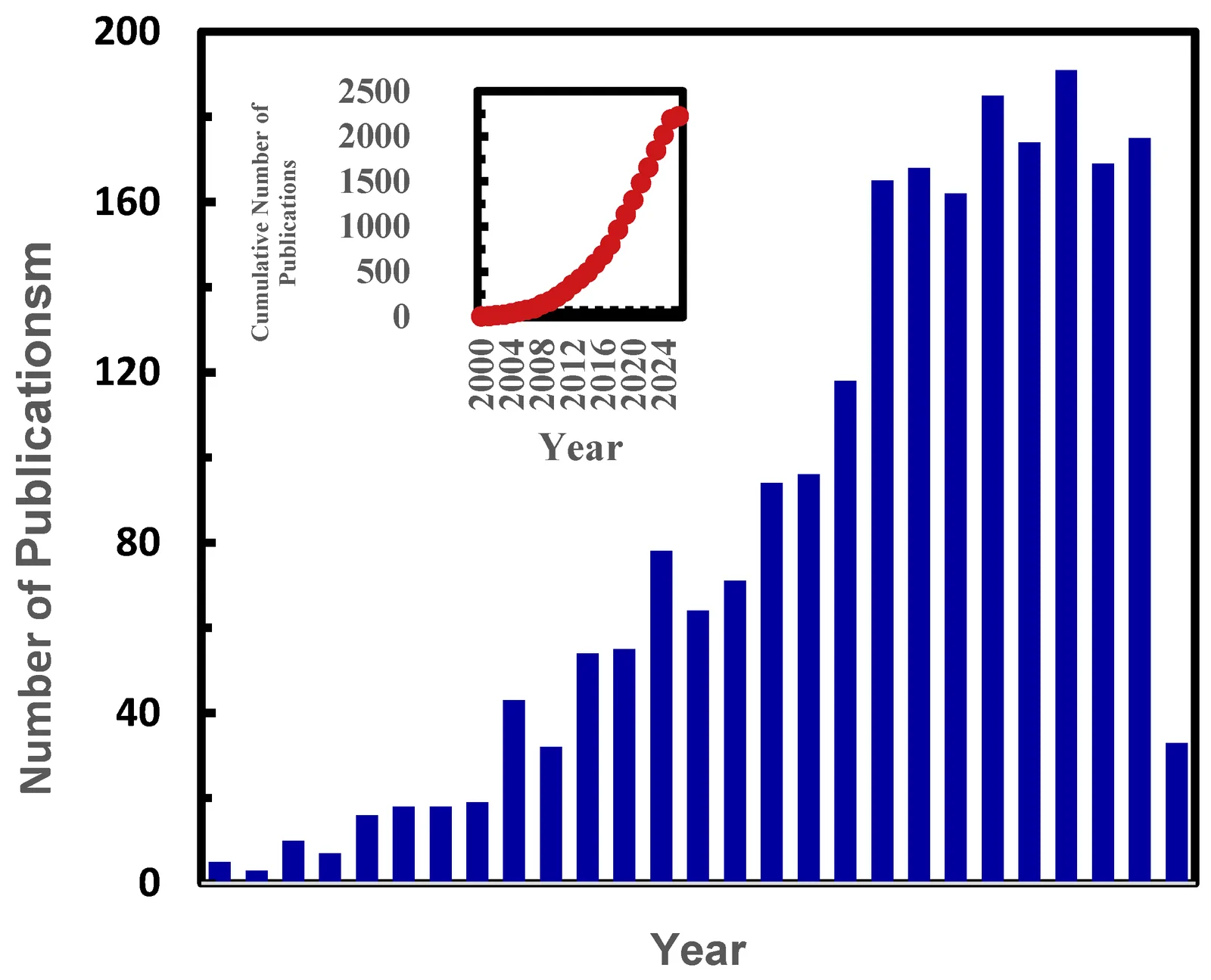
The number of papers published on *Geobacter*-related environmental research is 2223 from January 2000 to February 2026. The number of papers published has steadily increased over time. As research progresses, it receives increasing attention in environmental applications. *Geobacter*-related environmental research can be divided into two distinct periods: the “initial accumulation period” 2000–2010 and the “rapid growth period” 2011–2026.

Analysis of annual publication distribution serves as a fundamental component of bibliometric research. Statistical quantification of publication outputs within a specific timespan can intuitively reflect the developmental trajectory and dynamic changes in academic attention in a given research field. This study systematically analyzed the annual publication trends and cumulative growth patterns of global *Geobacter* and *Trichlorobacter* research from 2000 to 2026, revealing a two-stage evolutionary characteristic involving initial gradual emergence and subsequent rapid expansion. From 2000 to 2010, the annual publication volume increased moderately with a low average annual output, indicating that this decade corresponded to the preliminary exploratory and foundational development stage of the research field. Since 2011, the number of annual publications has increased significantly, and the growth trend has further accelerated after 2015, with continuous record-breaking annual outputs. By 2024, the annual publication volume neared 200, demonstrating a sustained rise in global academic interest in *Geobacter*-related research. This progressive upward trend is driven by multiple practical and scientific factors. On the one hand, global ecological problems such as heavy metal pollution and refractory organic contamination have become increasingly severe, while conventional remediation technologies face prominent limitations including high economic costs and technical bottlenecks. On the other hand, a series of pivotal scientific breakthroughs achieved after 2011—including the systematic clarification of extracellular electron transfer mechanisms, the identification and functional characterization of microbial nanowires, and the verification of interspecies electron transfer behaviors—have strongly promoted the in-depth development of this field. Based on the quantified annual growth rates and temporal dynamic characteristics, the overall development of *Geobacter* research can be divided into two distinct stages: the initial accumulation stage (2000–2010) and the rapid growth stage (2011–2026). This phased chronological framework provides a reliable basis for further exploring the evolutionary rules of research themes and hotspots across different periods (Figure 2).

### 3.1. Core Authors and High-Impact Research Groups

Author bibliometric analysis is conducive to identifying core research contributors and dominant research focuses within a specific academic domain, which helps elucidate the scholarly composition and academic collaboration network structure of the research field. From 2000 to 2026, numerous researchers have contributed to the advancement of *Geobacter* functional mechanisms and environmental remediation research. Table 1 summarizes three core statistical results, including the top 10 productive authors ranked by publication quantity, the top 10 highly cited authors ranked by total citation counts, and the top 10 high-yield journals in this research field.

In terms of publication volume, the top ten most productive authors are primarily affiliated with institutions in the United States, China, and Europe, forming a global research pattern centered on the United States, with Europe and Asia as essential regional nodes. Researchers from the United States contribute the largest share of publications, highlighting its longstanding dominant position in this field. Notably, several leading authors belong to the same research institutions, demonstrating the formation of stable and cooperative core research groups supported by sustained project investment and long-term strategic research planning. Their research covers the full spectrum of this discipline, ranging from foundational investigations of microbial physiology and biochemistry to the engineering optimization and practical deployment of bioelectrochemical systems, reflecting good systematic integrity and long-term research continuity.

In comparison, Chinese scholars are widely distributed across numerous domestic universities and research institutes, indicating extensive institutional participation and vigorous research engagement in *Geobacter*-related studies. In terms of research orientation, Chinese researchers predominantly focus on applied research, including system optimization, scale-up implementation, and practical remediation engineering, which effectively complements the mechanism-oriented foundational studies dominated by European and American scholars.

European research teams mainly concentrate on the biophysical mechanisms of extracellular electron transfer at molecular and nanoscales. Such research features prominent interdisciplinary attributes, integrating the theoretical and methodological advances in microbiology, electrochemistry, biophysics, and materials science.

In terms of citation performance, highly influential authors exhibit obvious regional clustering characteristics. The top ten highly cited authors are overwhelmingly affiliated with U.S. institutions. Most of these leading scholars maintain close academic connections, including stable mentorship inheritance and long-term collaborative relationships, forming a cohesive academic community with continuous knowledge accumulation and disciplinary inheritance. Their research focuses on three core and interconnected directions: (i) extracellular electron transfer mechanisms of electroactive microorganisms; (ii) design, optimization, and engineering applications of bioelectrochemical systems; and (iii) discovery and experimental verification of interspecies electron transfer pathways. These core research directions constitute the fundamental theoretical framework and knowledge basis of the discipline.

Although European and Asian scholars have gradually appeared in high-citation rankings, their overall number and average citation indicators remain relatively inferior to those of U.S. researchers. A clear discrepancy exists between publication volume rankings and citation impact rankings. Some scholars produce fewer publications but achieve extremely high citation levels, indicating the high originality, academic influence, and universal recognition of their findings. In contrast, while Chinese scholars show prominent advantages in publication quantity, their representation in high-impact citation rankings remains limited. This disparity suggests that although China has formed a large-scale and comprehensive research foundation in this field, further breakthroughs in theoretical innovation and disciplinary leading research are still required.

Overall, the global research pattern of *Geobacter* studies features coordinated development of foundational theories and applied technologies. The United States and Europe act as the core intellectual hubs driving disciplinary theoretical progress, while Asian countries witness rapid research growth with increasing academic influence. Continuous national research investment and in-depth international academic collaboration are expected to further reshape the global development landscape of this field in the future.

### 3.2. Cross-Disciplinary Characteristics and Distribution of Research Fields

**Figure 3:**
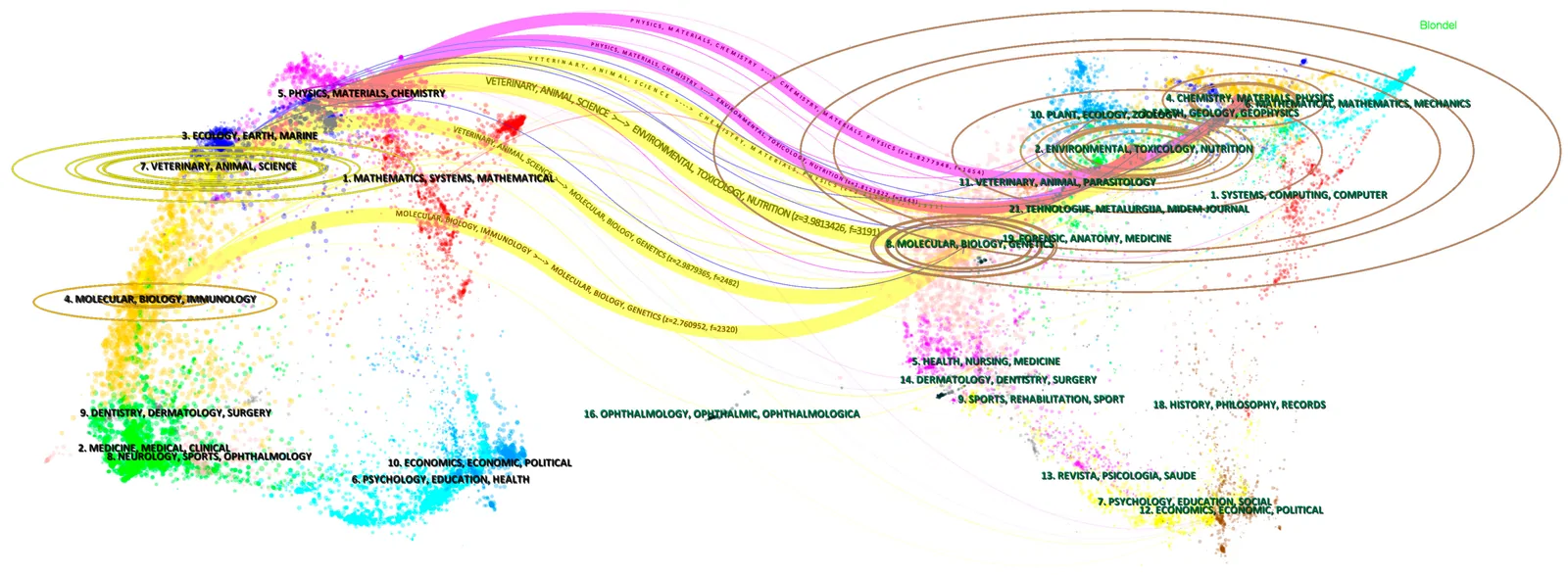
Dual-map overlay analysis of *Geobacter* research. The left panel illustrates the subject distribution of the source literature. *Geobacter*-related research mainly covers fields such as chemistry and physics. The right panel shows the subject distribution of cited literature, which is predominantly concentrated in disciplines including ecology and environmental science, reflecting the multidisciplinary nature of this research field.

Dual-map overlay analysis is a robust bibliometric approach to characterize disciplinary distribution, citation trajectories, and thematic concentrations of academic publications, which can intuitively visualize the intrinsic interdisciplinary correlations and cross-cutting attributes of a specific research domain. The dual-map overlay results of global *Geobacter* research are presented in Figure 3, where the left panel illustrates the thematic distribution of citing literature and the right panel reflects the disciplinary distribution of cited literature.

The left panel indicates that the thematic framework of citing literature forms a three-layered research paradigm dominated by biology, oriented toward environmental science, and supplemented by materials science. Biology serves as the fundamental disciplinary foundation, supporting core mechanistic investigations covering microbial metabolism, extracellular electron transfer processes, and pollutant degradation pathways. Meanwhile, environmental science and ecology orient relevant research toward practical environmental governance demands, constructing a complete translational research chain from laboratory-based mechanistic exploration to field-scale remediation application.

In recent years, the interdisciplinary integration of materials science and chemistry has become a critical developmental trend in this field. With the rapid innovation of bio-electrochemical remediation technology, the design and fabrication of high-performance electrode materials, as well as the functional modification and structural regulation of nanomaterials, have evolved into prominent research hotspots. Specifically, materials science provides theoretical guidance for optimizing the structural configuration and interfacial reaction processes of bioelectrochemical systems, while chemistry offers essential technical support for regulating electrode surface properties, reaction activity, and microbial biocompatibility. Such multidisciplinary synergies have effectively broken through the inherent technical limitations of traditional microbial remediation strategies. This integration not only broadens the theoretical connotation and methodological boundaries of environmental biotechnology but also promotes a critical research paradigm shift from single-strain-based remediation to material–microorganism synergistic enhancement. This progress substantially improves the theoretical robustness and engineering scalability of *Geobacter*-based remediation technologies.

The disciplinary distribution of cited literature (right panel) further demonstrates that *Geobacter* research has established an open, extensive, and highly interconnected interdisciplinary knowledge network. In addition to relying on the foundational theories and technical methods of environmental science, microbiology, and materials chemistry, this field has increasingly absorbed cutting-edge theories and technologies from multiple adjacent disciplines, including computer science (e.g., machine learning-assisted material screening and process simulation) and geochemistry (e.g., solid–liquid interfacial reactions and redox speciation analysis). This feature verifies that bio-electrochemical remediation research is inherently transdisciplinary rather than a single independent research field. It develops as a dynamic and integrated research system that continuously incorporates innovative theories, computational tools, and experimental technologies from diverse disciplines.

Collectively, dual-map overlay analysis confirms that contemporary *Geobacter* research has formed a mature multidisciplinary system with biological mechanisms as the core, environmental application as the research orientation, and materials innovation as an important driving force. Cross-disciplinary integration constitutes a core engine for the sustainable innovation of this field and lays a solid foundation for the continuous emergence of new theories, methods, and technologies. With the gradual blurring of traditional disciplinary boundaries, this integrative developmental trend will become increasingly prominent, facilitating the development of more efficient, cost-effective, and eco-friendly environmental remediation and bioenergy technologies.

### 3.3. Analysis of the Country and Institutions

Bibliometric statistical analysis of author affiliations across countries can objectively reflect the national research capacity and global academic influence in *Geobacter* research. To date, researchers from 71 countries worldwide have contributed to this field, yet significant disparities exist in national research investment and publication output. To systematically clarify the global spatial pattern of *Geobacter* research, this study focuses on the top three productive countries. By analyzing the spatial distribution of research outputs and inter-institutional collaborative patterns, this study provides empirical insights for identifying emerging trends and core frontiers in this research domain.

**Figure 4:**
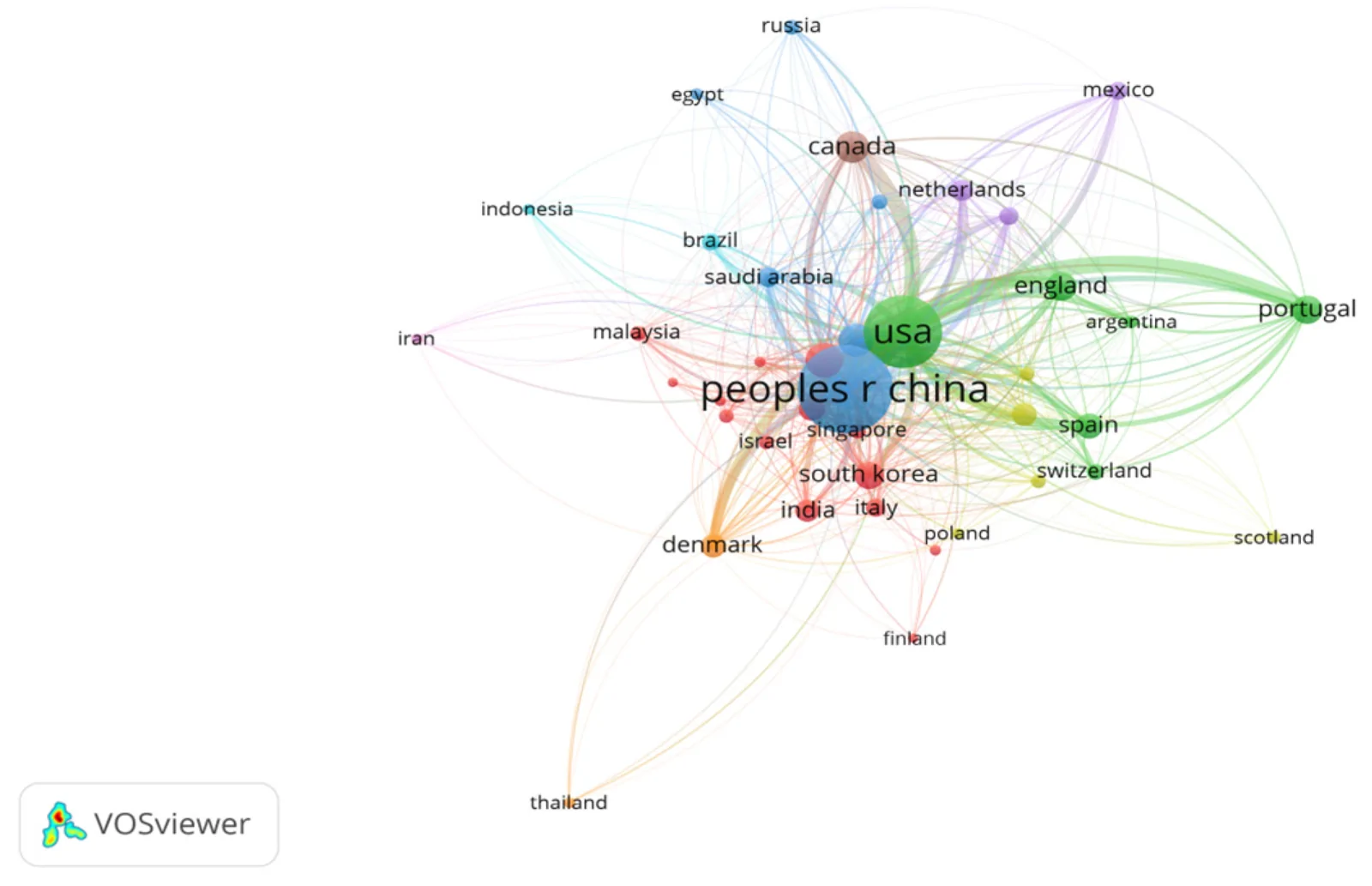
National distribution of *Geobacter*-related publications.

**Figure 5:**
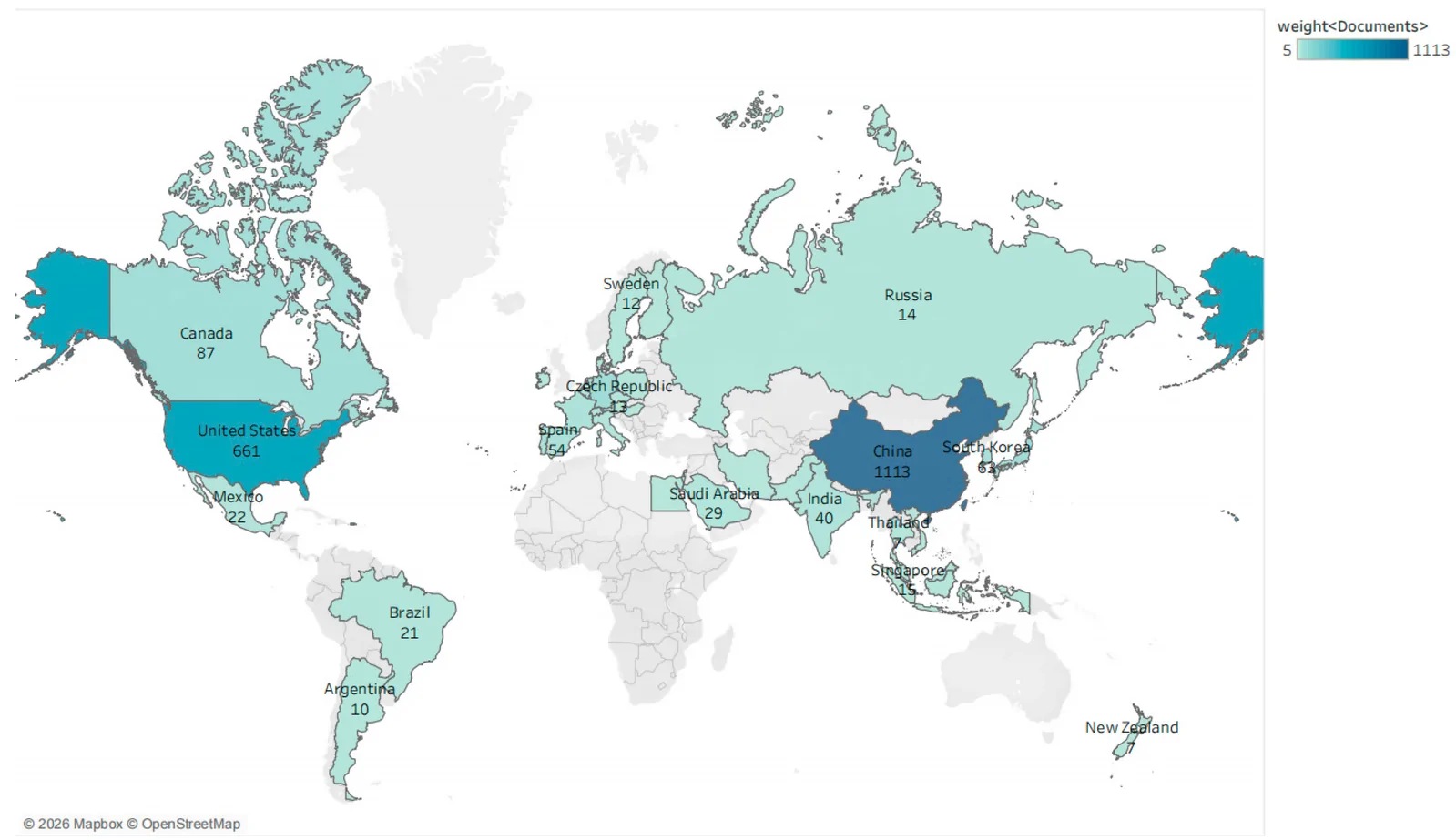
National publication contribution to *Geobacter* research.

According to the regional publication heatmap, China ranks first globally with 1113 publications, followed by the United States (661 publications) and Germany (110 publications). The cross-national collaborative links visualized in Figure 4 intuitively characterize the bilateral cooperation network of global *Geobacter* research. Benefiting from its large publication output and abundant applied research scenarios, China has developed into the largest global collaborative hub in this field. The United States maintains a core leading position through its dominant foundational and mechanistic research, while European countries—represented by Germany—provide key theoretical support and technical innovation via sustained and high-intensity international cooperation (Figure 5).

In future research, deepening strategic cooperative partnerships with leading research powers, including the United States and Germany, while expanding inclusive collaborative exchanges and capacity-building cooperation with developing countries will effectively enhance the overall innovation capacity of this field and further strengthen the global applicability and influence of *Geobacter*-based environmental remediation technologies.

Publication quantity is a direct and reliable indicator for evaluating the research strength and academic influence of individual institutions in a specific field (Table 2). Institutional analysis reveals that China occupies a dominant position in global *Geobacter* research, with six of the top ten high-output institutions originating from China. In terms of single-institution performance, the University of Massachusetts (USA) ranks first with 200 publications, followed by the Chinese Academy of Sciences (172 publications) and Harbin Institute of Technology (134 publications). Overall, *Geobacter* research presents a distinctive multinational and collaborative research landscape. Leading institutions from China and the United States occupy core strategic positions, highlighting the joint leadership of top research bodies and the interdisciplinary, collaborative nature of global research progress in this field.

### 3.4. Author and Co-Citation Analysis

**Figure 6:**
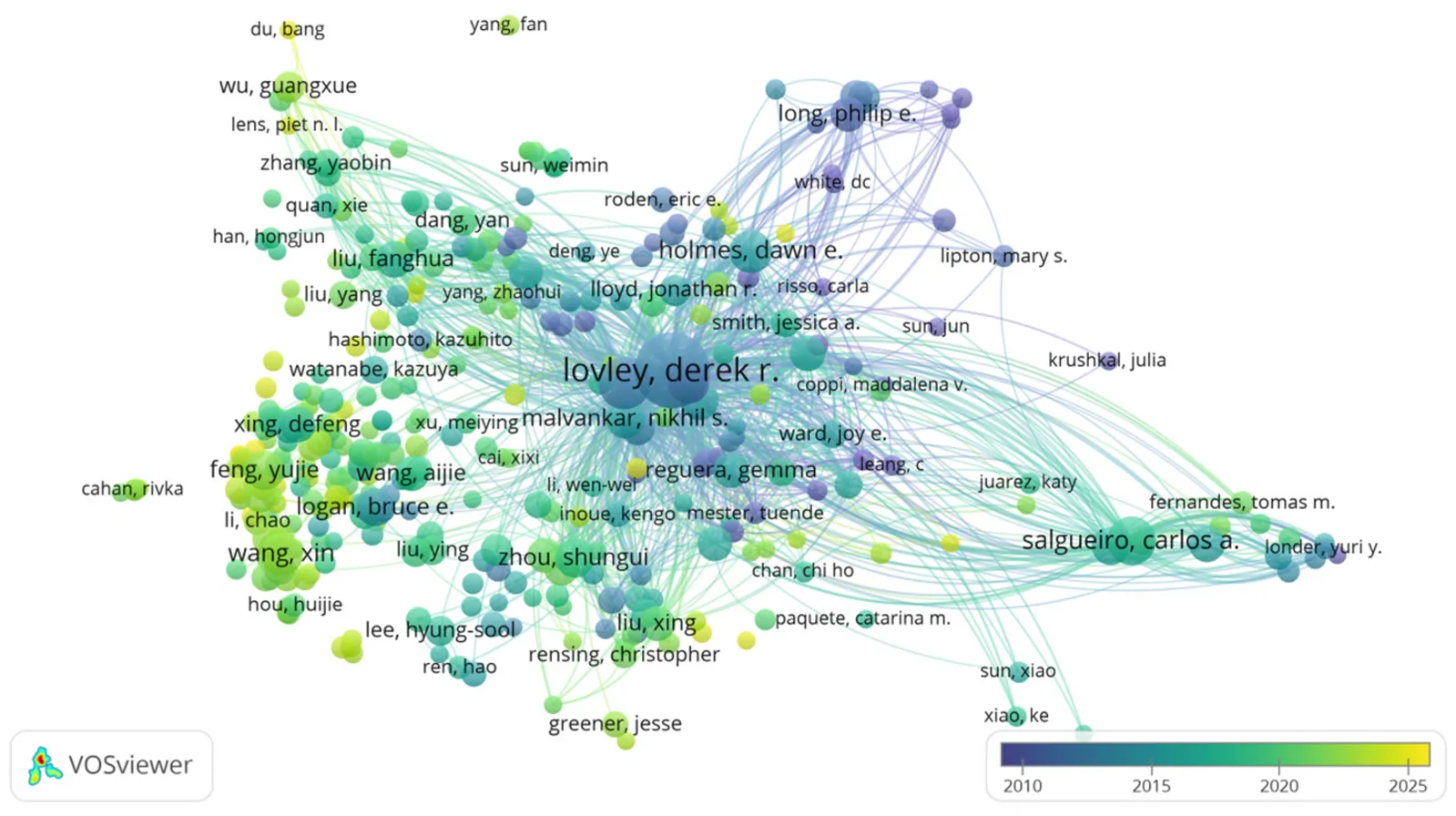
Author distribution and collaboration relationships.

In bibliometric studies, publication output reflects researchers’ persistent engagement and productive capacity in a specific domain, whereas citation frequency objectively quantifies peer recognition and the intrinsic academic value of their work. As two core complementary quantitative indicators, publication volume and co-citation frequency are widely employed to comprehensively evaluate scholars’ academic performance and disciplinary influence. Author network analysis enables the identification of pivotal contributors and dominant research priorities, which facilitates targeted academic cooperation and interdisciplinary communication. Additionally, such analysis provides credible indirect evidence for assessing the relative research competitiveness of different countries and regions. From 2000 to January 2026, a total of 7960 scholars across diverse disciplines have participated in global *Geobacter*-related research. As visualized in Figure 6, *Geobacter* research forms a highly compact and interconnected collaborative network with robust academic linkages among core researchers. Notably, Professor Derek R. Lovley is identified as the most productive and collaboratively active scholar, acting as the core nodal figure in the global author cooperation network of this field.

**Figure 7:**
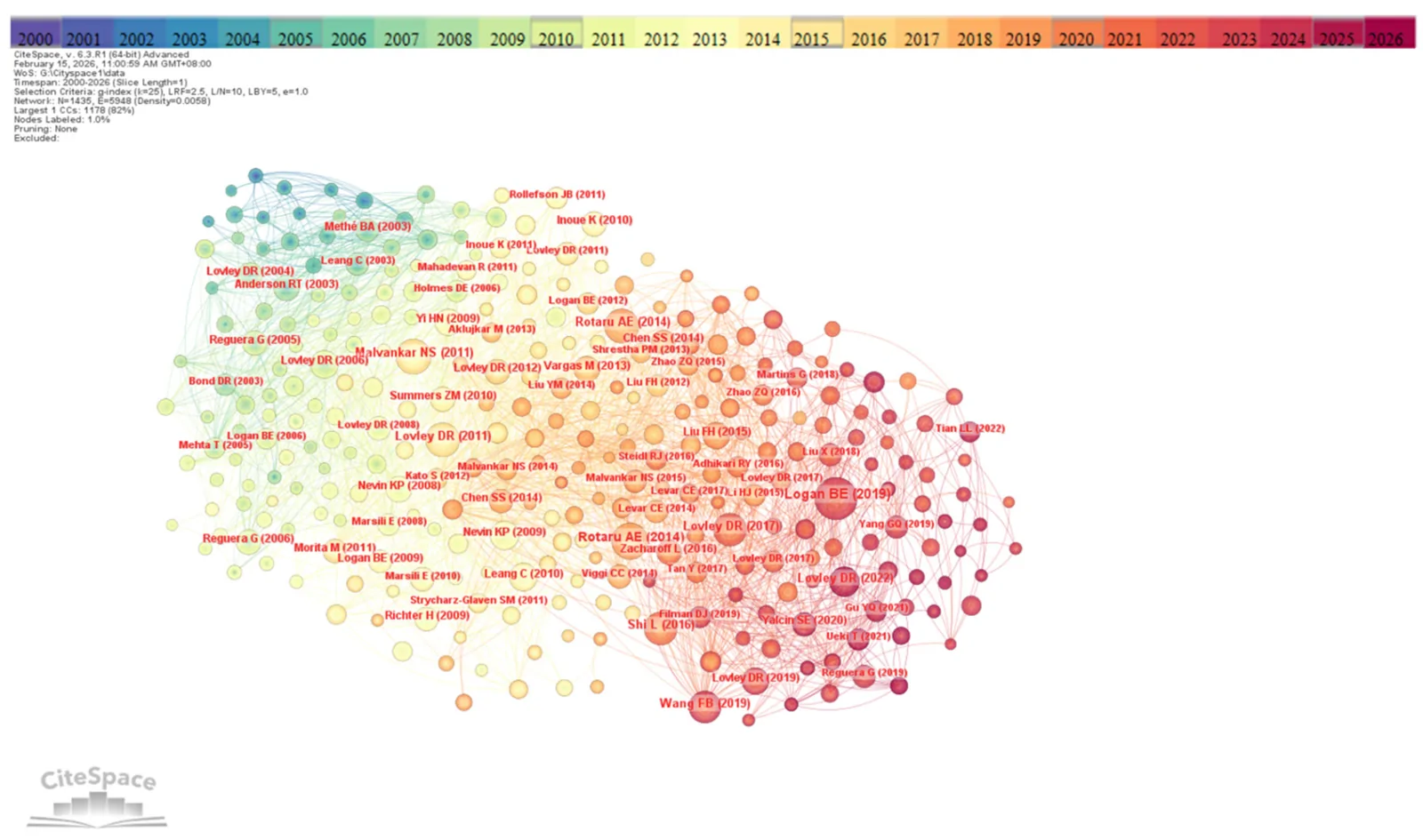
Co-cited literature. Each node represents a highly cited author, and the color of the node represents the time interval when the author was first cited, corresponding to the top time bar January 2000–February 2026.

Co-citation analysis serves as a vital bibliometric tool for identifying landmark achievements and core thematic frameworks that drive disciplinary development (Figure 7). Highly co-cited publications generally represent foundational theories, mainstream technical methodologies, and authoritative research paradigms within a given field. In this study, the co-citation network visualization includes authors with more than ten co-citation records. Among all contributors, Logan, B. E. and Rotaru, A. E. exhibit co-citation counts exceeding 100, ranking them as the most highly co-cited scholars in *Geobacter* research. Their studies primarily focus on the extracellular electron transfer mechanisms of *Geobacter* species and the innovative iteration of supporting bioelectrochemical technologies, further verifying that these topics constitute the central mainstream research themes of the domain.

### 3.5. Evolution of Research Hotspots and Popular Topics

**Figure 8:**
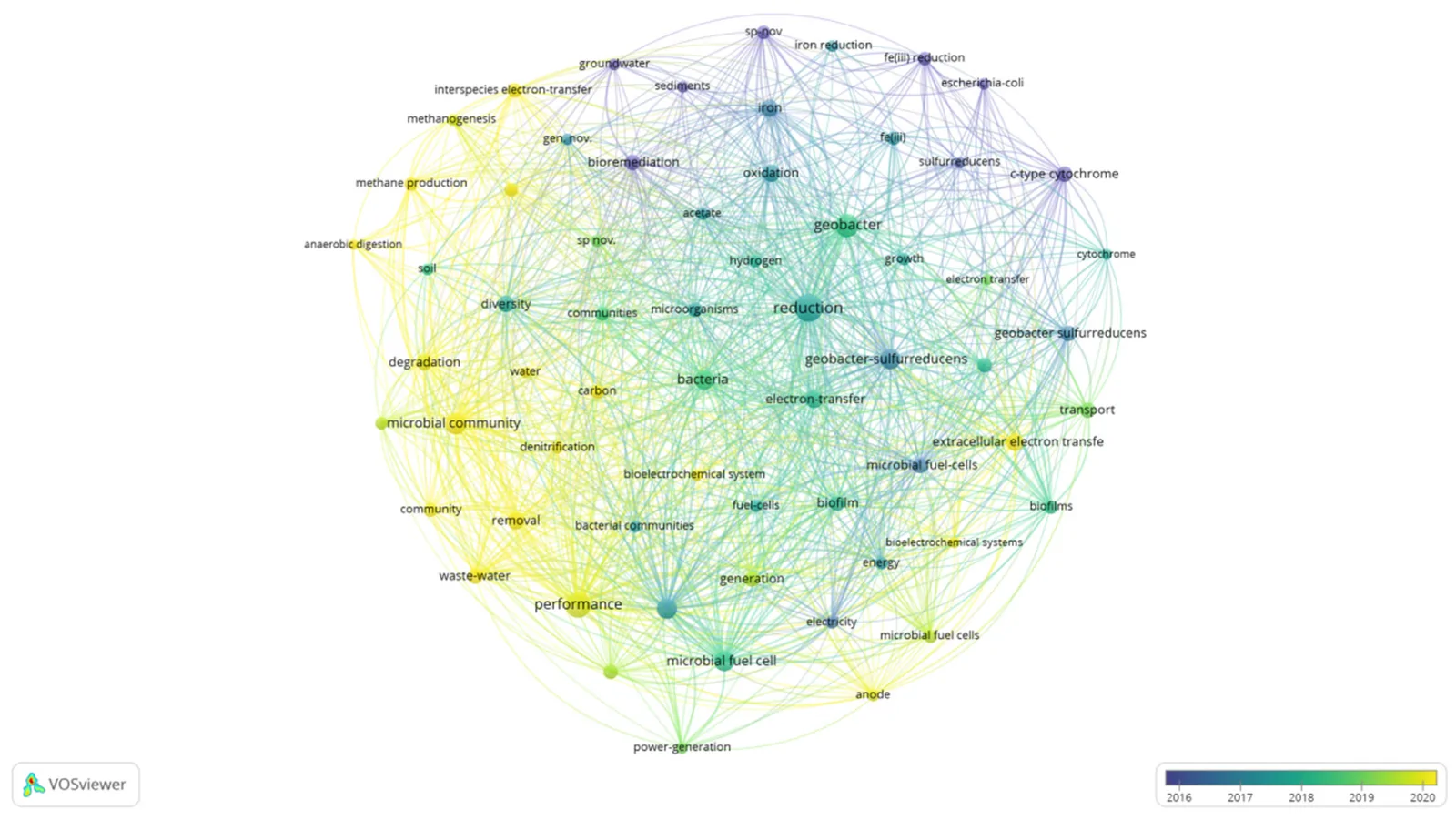
Keyword co-occurrence network: Each node represents a keyword, with its size proportional to the keyword’s occurrence frequency. Links between nodes indicate the co-occurrence relationship between paired keywords; the thicker the link, the stronger the co-occurrence association between the two keywords.

**Figure 9:**
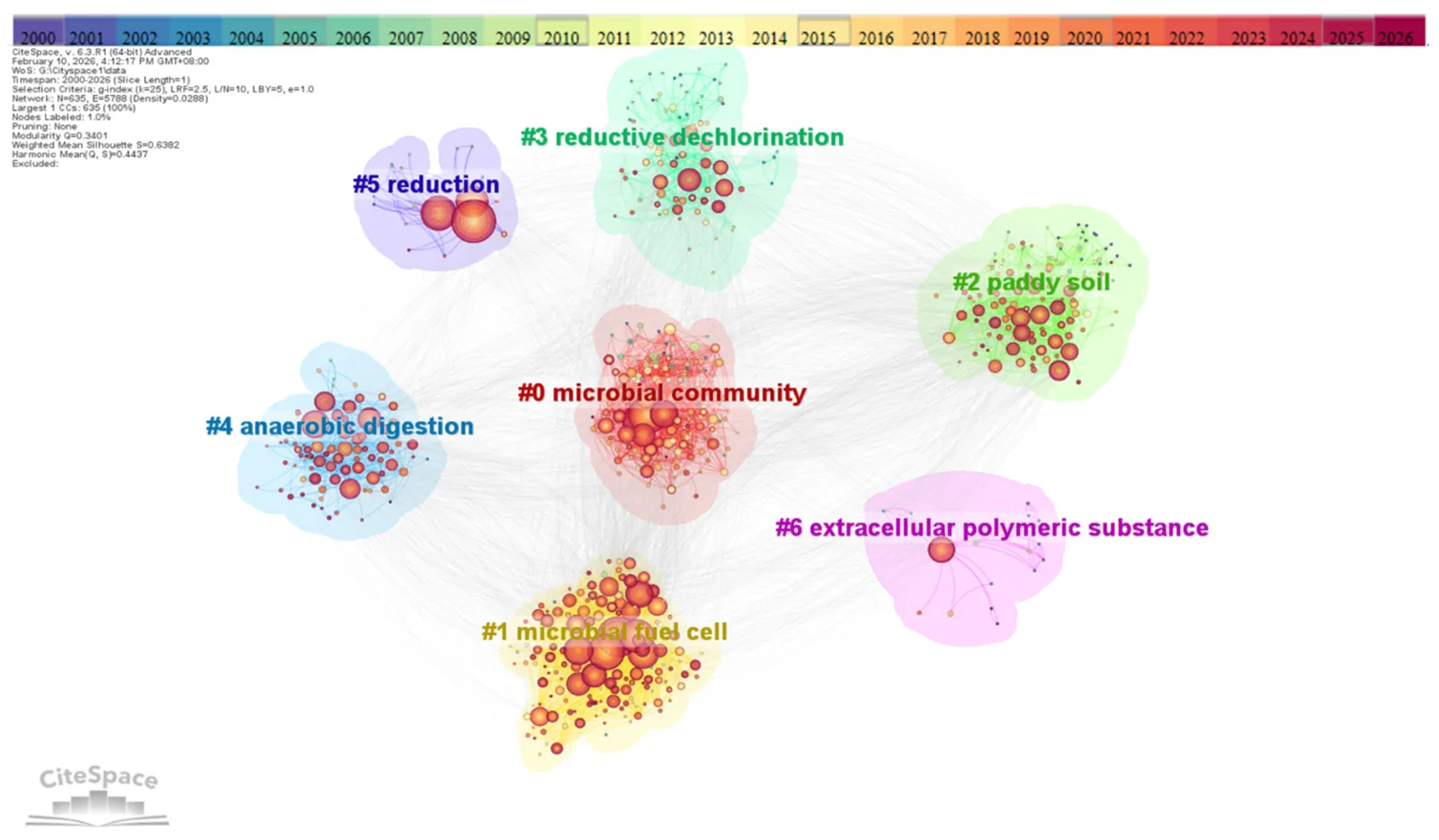
Keyword cluster nodes represent keywords, and their size is proportional to the frequency of the keyword. Regions with different colors indicate different research sub-directions, and the color of nodes and cluster labels is consistent to indicate their affiliation.

**Figure 10:**
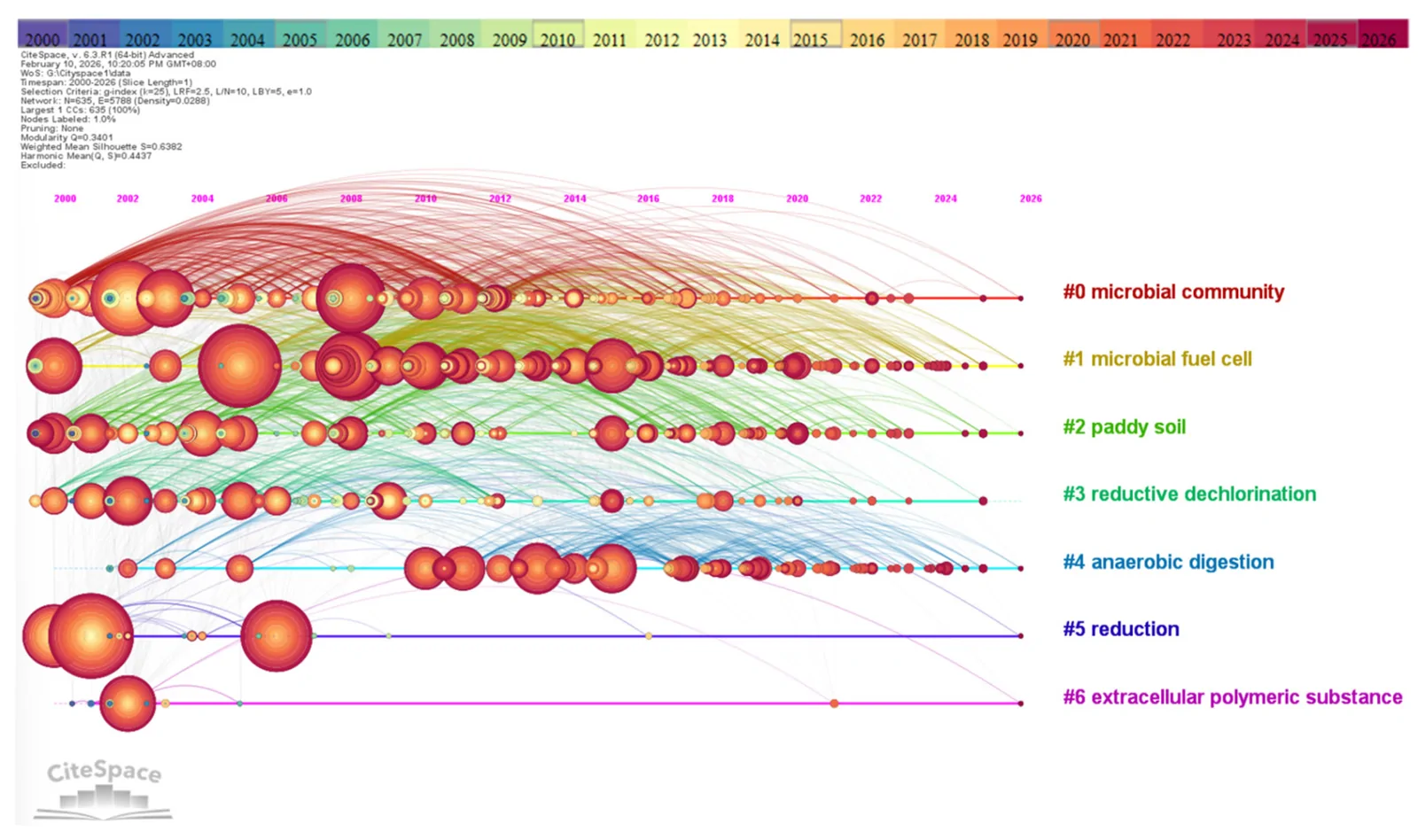
Keyword timeline view. The horizontal timeline represents years from left to right, and each vertical row corresponds to one keyword cluster.

Keyword-based bibliometric analysis is an effective approach to identify core research themes, track thematic evolutionary trajectories, and capture emerging disciplinary frontiers, as keywords precisely condense the key scientific questions and research focuses of the published literature. In keyword network visualization, each node represents an individual keyword, with node size proportional to occurrence frequency. The color and inner ring thickness reflect temporal dynamic variations in keyword popularity, while connecting edges indicate co-occurrence relationships, where edge thickness corresponds to the association intensity between paired keywords. This study integrated four core keyword-based analytical outputs to comprehensively decode the thematic evolution of *Geobacter* research: the keyword co-occurrence network (Figure 8) characterizes the association patterns of core research topics; the keyword clustering map (Figure 9) identifies dominant thematic clusters and their internal logical correlations; the keyword timeline view (Figure 10) elaborates the stage-specific evolutionary pathways of research hotspots; and the keyword burst detection analysis screens the top 25 high-burst keywords to pinpoint nascent and fast-growing research frontiers.

Synthesizing the above visualization results, bioremediation, *Geobacter*, reduction, and performance are identified as the perennial core research topics dominating this field. Overall thematic evolution reveals a clear disciplinary transition: early-stage studies prioritized fundamental ecological and environmental mechanism exploration, while recent research has gradually shifted toward applied and comprehensive research oriented toward sustainable environmental governance. Seven major thematic clusters were classified in this domain, including #0 microbial community, #1 microbial fuel cell, #2 paddy soil, #3 reductive dechlorination, #4 anaerobic digestion, #5 reduction, and #6 extracellular polymeric substances. Notably, Cluster #0 (microbial community) acts as the central and most productive research module, possessing extensive interconnections with all other subfields and supporting the overall development of *Geobacter* research.

The temporal evolution of burst keywords further demonstrates distinct stage-specific research characteristics. In the initial developmental stage, the research community primarily focused on microbial community structure and microbial reduction mechanisms. Highly bursted keywords, including Escherichia coli (intensity = 14.98), dissimilatory Fe(III) reduction (13.43), uranium (12.69), and metal reduction (10.45), highlight the dominant research priority of exploring microbial reduction principles and heavy metal remediation strategies during this period. In the intermediate developmental phase, research scope expanded substantially toward electroactive microbial technologies and ecological remediation systems. The prominent burst ofc-type cytochrome (11.22), microbial fuel cell (10.08), and constructed wetland (9.64) indicate a clear research transition from basic mechanistic exploration to extracellular electron transfer mechanisms, ecological engineering, and bioelectrochemical application, representing obvious thematic diversification and application-oriented expansion.

**Figure 11:**
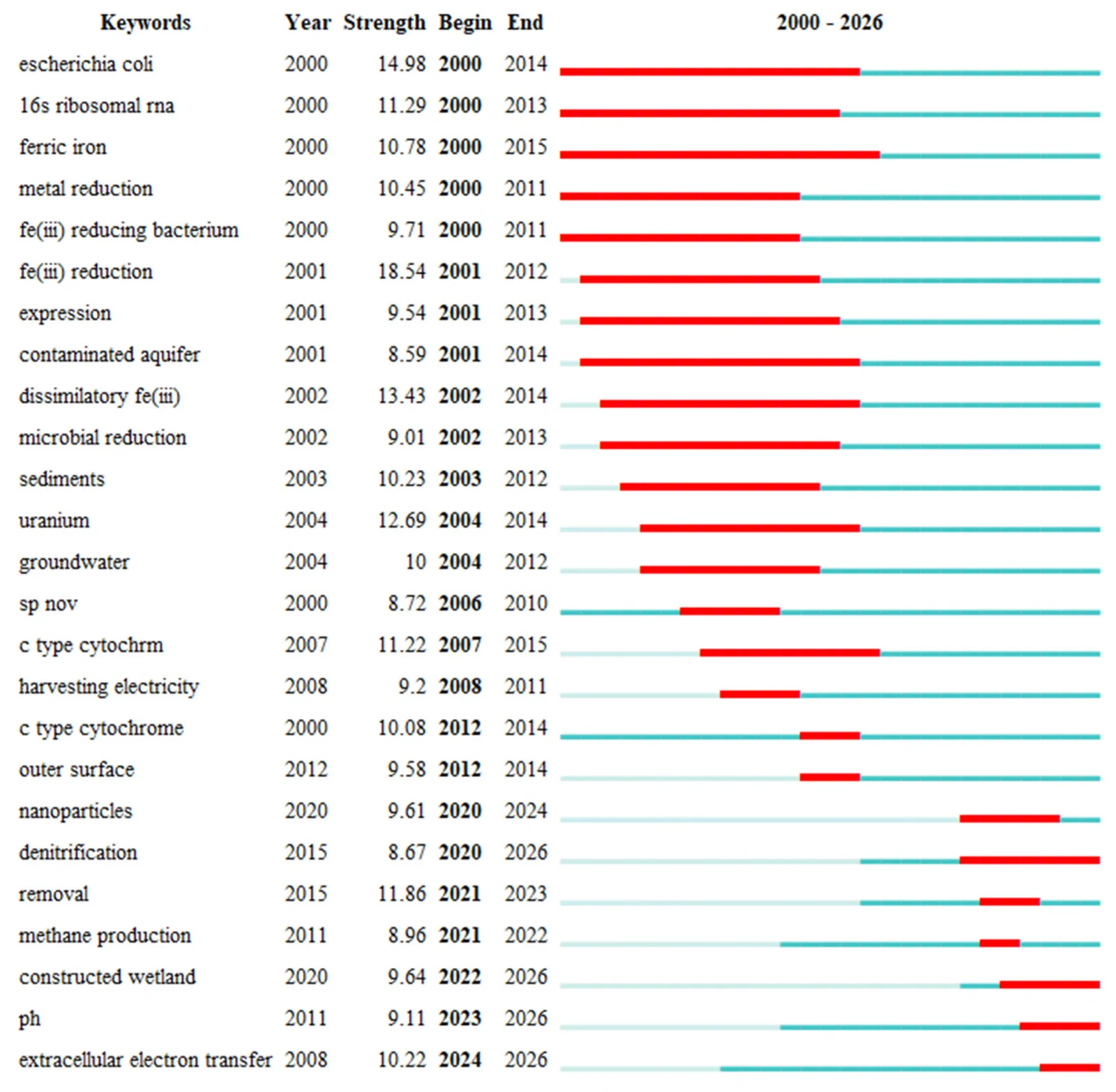
Keyword burst detection map. The 25 keywords with the highest burst strength are listed on the left. The red segments mark the start and end years of each keyword’s burst period, while the blue segments indicate the overall duration of the keyword across the full study period before and after the burst.

In recent years, research concerning extracellular polymeric substances and interfacial microbial processes has remained highly active. Newly emerging high-burst keywords, including removal (11.86), extracellular electron transfer (10.22), nanoparticles (9.61), and denitrification (8.67), reflect the latest evolving frontiers of this field (Figure 11). These trends demonstrate that current research increasingly focuses on nanomaterial-assisted pollution remediation, nitrogen cycle regulatory mechanisms (particularly denitrification processes), and in-depth interpretation of EET interfacial reaction mechanisms. Furthermore, persistent burst keywords including extracellular electron transfer (2008–2026), pH (2023–2026), and denitrification (ongoing to 2026) maintain continuous growth momentum, strongly confirming their status as the most promising and sustained research frontiers guiding future *Geobacter*-related disciplinary development.

## 4. Discussion

The genus *Geobacter* is ubiquitously distributed in natural and engineered environmental systems. As a metabolically versatile electroactive microbial group, *Geobacter* species possess the unique capability of mediating direct extracellular electron transfer (EET) toward diverse exogenous electron acceptors, including metal oxides, conductive electrodes, and adjacent microbial cells [19]. Such distinctive physiological traits endow this genus with crucial regulatory functions in environmental biogeochemical cycling and immense application potential in environmental biotechnology [20]. Notably, sulfur-reducing *Geobacter* strains serve as classic model organisms for unraveling the molecular and physiological mechanisms governing EET processes within the genus [12]. As predominant high-efficiency iron-reducing microorganisms, *Geobacter* species occupy a core nodal position in global biogeochemical iron cycling. Beyond their fundamental ecological functions, they contribute indispensably to wastewater purification, microbial fuel cell (MFC) bioenergy conversion, and nanowire-based bioelectronic development, demonstrating broad disciplinary coverage and dual value in basic theoretical research and practical engineering applications [21,22].

This study conducted a systematic bibliometric analysis based on the Web of Science Core Collection, targeting peer-reviewed publications related to *Geobacter* and *Trichlorobacter* from 2000 to 2026, with a core focus on bioremediation, iron reduction, microbial fuel cells, and biogeochemical cycling. After rigorous manual screening and duplicate removal, a total of 2223 valid articles were included for subsequent multidimensional analysis. The analytical framework covered author contribution characteristics, keyword co-occurrence evolution, journal distribution, national and institutional research output, and citation network relationships. This comprehensive bibliometric assessment systematically clarified the 25-year developmental trajectory of global *Geobacter* research and objectively identified dominant thematic hotspots and emerging future frontiers. The results reveal that global scholarly attention has long converged on two core research clusters: one centered on bioelectrochemical systems (represented by MFCs) based on the unique EET properties of *Geobacter*, and the other focused on environmental remediation and wastewater treatment relying on its versatile respiratory metabolic functions. Accordingly, extracellular electron transfer mechanisms, microbial fuel cell technologies, biogeochemical cycling processes, and their interdisciplinary intersections will continue to dominate the research frontier of this field in future investigations.

### 4.1. Extracellular Electron Transfer (EET)

Extracellular electron transfer serves as the most fundamental and persistent core research theme in *Geobacter* biology. As a unique anaerobic respiratory physiological process, EET enables *Geobacter* species to transfer intracellular electrons to outer-membrane and extracellular environments, thereby utilizing exogenous insoluble electron acceptors to sustain metabolic activity. Consistent with keyword co-occurrence and burst detection results, EET maintains the highest centrality and longest research duration throughout the developmental history of this domain [23]. This is mainly driven by the growing practical demands for in situ pollution remediation in global anaerobic environments and the development of microbial electrochemical energy. As the core physiological basis enabling pollutant reduction and chemical-to-electrical energy conversion in *Geobacter*, EET efficiency directly sets the application ceiling of related technologies, making it a key research target to break engineering bottlenecks. Meanwhile, advances in multi-omics and other technologies have provided powerful tools for unraveling the electron transfer mechanisms of c-type cytochrome networks and conductive pili, shifting research in this field from macroscopic physiological phenotypic observation toward mechanistic elucidation at molecular and atomic scales.

*Geobacter sulfurreducens* is universally recognized as the canonical model strain for EET mechanism research, and quantitative characterization of its EET kinetic parameters is essential for advancing the iterative optimization of microbial electrochemical technologies [24]. This study further refines the kinetic quantification of core EET processes in *G. sulfurreducens*, providing refined parameter support for subsequent mechanism exploration and technological upgrading [25].

Bioelectrochemical systems (BESs) represent a sustainable and integrated biotechnology platform that achieves simultaneous wastewater remediation and renewable energy recovery. The efficient bidirectional electron exchange between electroactive microorganisms and electrode interfaces determines the overall operational performance of BESs [26]. This study identified and validated a novel regulatory pathway whereby artificial electron mediators effectively enhance the EET efficiency of *Geobacter* species, offering theoretical guidance and actionable strategies for the rational design and optimized application of electron mediators to improve BES operational efficiency [27].

From a temporal evolutionary perspective, early-stage EET research primarily focused on macroscopic phenotypic observation and basic functional verification. Early studies concentrated on exploring the core roles of extracellular cytochromes, conductive pili, and other redox-active substances in mediating electron transfer to terminal acceptors such as Fe(III) oxides and heavy metal pollutants. Distinct from most heterotrophic microorganisms, *Geobacter* species deploy abundant multiheme c-type cytochromes across the inner membrane, periplasmic space, outer membrane, and extracellular microenvironment. These functionally differentiated cytochrome systems constitute a complete electron transport chain, enabling efficient EET with diverse electron donors and acceptors [28]. Additionally, nanoscale magnetite particles have been verified to facilitate microbial EET processes, playing a vital regulatory role in biogeochemical cycling, in situ bioremediation, and bioenergy engineering. The structural and functional integration between magnetite nanoparticles and *Geobacter* EET machinery is analogous to the coupling mode between the polyheme cytochrome OmcS and conductive pili in *G. sulfurreducens*, further explaining the intrinsic mechanism of nanomaterial-mediated EET enhancement [29].

With the widespread application of multi-omics technologies in microbiological research, medium-stage EET investigations gradually transitioned from phenomenological description to molecular-level mechanistic dissection, substantially deepening the systematic understanding of EET pathways and endogenous regulatory networks. In recent years, the synergistic interaction between nanomaterials and microbial EET has emerged as a cutting-edge research hotspot. Specifically, the conductive nanowires secreted by *G. sulfurreducens* have become a paradigmatic research model for long-range extracellular electron transfer. These microbial nanowires are increasingly regarded as sustainable green functional nanomaterials, possessing transformative application potential in bioelectronic device fabrication, renewable energy conversion, and advanced environmental remediation. Multiple genetic modification, biochemical regulation, and environmental optimization strategies have been successfully developed to modulate the synthesis and functional expression of microbial nanowires [30].

Unlike conventional microorganisms that rely solely on intracellular soluble electron acceptors for respiration, *Geobacter* species can deliver intracellular electrons to extracellular insoluble substrates via multiple independent pathways, which is the core physiological basis for their superior performance in bioelectrochemical systems and environmental bioremediation [31]. To date, three major synergistic mechanisms have been systematically elucidated to dominate EET processes in *Geobacter*:

(1) Cytochrome-mediated direct electron transfer. *Geobacter sulfurreducens* acts as the standard model strain for characterizing direct EET pathways. A series of multiheme c-type cytochromes sequentially transfer electrons from intracellular metabolic pathways to extracellular substrates, including insoluble Fe(III) oxides and conductive electrode surfaces, realizing efficient trans-membrane electron transmission [32,33].
(2) Conductive pilus-based long-range electron transfer. Conductive pili, commonly defined as microbial nanowires, endow *Geobacter* biofilms with macroscopic electrical conductivity. The metallic-like conductive properties of *G. sulfurreducens* pili originate from π–π orbital stacking of internal aromatic amino acid residues [34]. Emerging evidence further confirms that *Geobacter* nanowires mediate direct interspecies electron transfer (DIET), a key syntrophic mechanism that facilitates the collaborative degradation of organic waste and methane production in anaerobic systems [35].
(3) Soluble redox shuttle-mediated indirect electron transfer. Using *G. sulfurreducens* as the model organism, previous studies have systematically characterized the functional properties of core outer-membrane cytochromes, including OmcS, OmcZ, and OmcB [36]. Nevertheless, a systematic theoretical framework integrating environmental signal induction, intracellular cascade regulation, and functional protein synergistic expression remains incomplete. Elucidating the multi-factor interactive regulatory network of EET is currently a core research frontier and a key breakthrough direction in *Geobacter mechanistic* research.

Research on EET in *Geobacter* not only enriches the theoretical framework of microbial anaerobic energy metabolism and expands academic understanding of extracellular biological processes, but also provides core theoretical support for various fields including in situ remediation of groundwater contamination, the development of microbial fuel cells, and the regulation of natural carbon, nitrogen, and iron cycles. It acts as a vital bridge linking fundamental microbiology and environmental engineering applications.

### 4.2. Microbial Fuel Cell (MFC)

*Geobacter* is recognized as a high-efficiency electroactive microbial genus with superior power generation density [37]. It can efficiently convert chemical energy stored in organic substrates into electrical energy, thus serving as the core biocatalyst for microbial fuel cell operation. MFCs are eco-friendly electrochemical devices that utilize microorganisms as natural biocatalysts to realize direct energy conversion from organic compounds to electricity. The core operational logic of MFCs lies in coupling microbial metabolic processes with extracellular electron transfer, achieving dual functions of pollutant degradation and energy recovery, which endows this technology with prominent environmental and economic advantages [38]. Against the dual pressures of global water scarcity and energy transition, the drawbacks of high energy consumption and carbon emissions inherent to conventional wastewater treatment technologies have become increasingly prominent. Characterized by simultaneous organic pollutant degradation and electricity recovery, microbial fuel cells (MFCs) precisely meet the demand for low-carbon wastewater resource recovery, emerging as a shared research hotspot in environmental engineering and bioenergy. Furthermore, the progressive elucidation of extracellular electron transfer mechanisms in *Geobacter* lays core theoretical foundations for MFC performance optimization. Particularly, the identification of direct electron transfer pathways enables targeted regulation of electron transfer efficiency in anode biofilms, shifting research focus from preliminary device exploration to mechanism-driven performance improvement.

Among all engineering applications of *Geobacter* EET functions, MFC technology represents the most mature and promising research direction [39,40,41]. Bibliometric results indicate that global MFC research centered on *Geobacter* is predominantly led by the United States, China, and European countries, forming a stable tripartite research pattern.

As a global pioneer in MFC basic research, the United States focuses primarily on mechanistic exploration and technological innovation, devoting extensive efforts to optimizing *Geobacter*-mediated energy conversion efficiency, developing high-performance electrode materials, and dissecting underlying electron transfer mechanisms. In this study, the potential for converting lignocellulosic biomass directly to electricity in an MFC was explored. In the absence of methanogenesis, bioaugmentation with *Geobacter metallireducens* further improved MFC performance [42]. Beyond direct power generation, MFCs and their derivative microbial electrolysis cells (MECs) can realize hydrogen energy recovery during organic matter degradation. 16S rRNA gene sequencing analysis consistently verifies that *Geobacter* species dominate the anodic functional microbial community of MECs, determining the core energy conversion performance of the system [43].

Chinese research in this field exhibits distinct application-oriented characteristics, focusing on expanding the engineering application scenarios of MFCs based on the unique metabolic advantages of *Geobacter* [44]. Centering on domestic practical demands for municipal wastewater treatment and organic waste resource utilization, Chinese scholars have developed diversified optimized MFC configurations for targeted pollution remediation. Driven by practical engineering needs, China has accumulated the largest volume of MFC-related publications globally, forming a research feature dominated by applied technological innovation and scenario expansion.

The power generation performance of MFCs relies heavily on the stable colonization and proliferation of electrogenic biofilms on anode surfaces. Therefore, exploring the electrochemical performance of anodic biofilms and the temporal succession characteristics of microbial communities is critical for MFC optimization. Previous studies have investigated MFC biofilm formation using three typical inoculum sources, including domestic wastewater, lake sediment, and biogas sludge. The results demonstrate that the model electroactive strain *Geobacter sulfurreducens* can be stably enriched in all inoculum systems. Furthermore, its relative abundance is significantly positively correlated with key MFC performance indicators such as current density and Coulombic efficiency, confirming its core functional status in MFC power generation [45].

In constructed wetland−microbial fuel cell (CW-MFC) coupled systems, anode filler material properties are the key determinants of nutrient removal efficiency and bioelectricity generation performance. To address the limitations of conventional fillers, an innovative pyrite-based CW-MFC (PCW-MFC) system has been developed and optimized. Microbial community analysis indicates that pyrite modification selectively enriches functional microorganisms associated with nitrogen and phosphorus removal, as well as electroactive *Geobacter* species. The synergistic effect between functional microbes significantly improves the comprehensive remediation and power generation performance of the system, providing theoretical support and technical reference for the rational design and performance optimization of advanced CW-MFC filler materials [46].

MFC technology enables synchronous organic pollutant degradation, wastewater purification, and resource recovery, exhibiting prominent comprehensive application value. In the remediation of emerging persistent pollutants such as tetrabromobisphenol A (TBBPA), synergistic microbial consortia composed of hydrolytic dehalogenators (*Acinetobacter*), aromatic degraders (*Geobacter*), and electrogenic bacteria (*Desulfuromonas*) play a decisive role in hydrolytic debromination and pollutant detoxification. The functional complementarity among different microbial taxa effectively enhances the remediation efficiency of refractory pollutants and broadens the application boundary of MFC technology in emerging environmental pollution remediation [47].

In terms of targeted strain optimization, metabolic engineering provides a novel technical pathway to further improve the electricity generation performance of MFCs. A genetically engineered *Geobacter* strain has been documented in a relevant patent: by introducing a functional mutation into the gene encoding the repressor of a key tricarboxylic acid (TCA) cycle enzyme, the strain achieves efficient utilization of pyruvate and 3–12 carbon pyruvate metabolic precursors as electron donors, with significantly expanded substrate utilization scope and enhanced metabolic efficiency. This strain can be directly deployed to augment the power output of microbial fuel cells, offering a solution rooted in endogenous metabolic regulation of strains to overcome the bottleneck of MFC energy conversion efficiency [48].

Despite the substantial progress achieved in *Geobacter*-based MFC research, multiple technical bottlenecks still restrict its large-scale industrial application. The unique EET capability of *Geobacter* biofilms endows MFCs with sustainable power generation potential; however, the stability, durability, and power density of *Geobacter* biofilm electrochemical performance under complex environmental conditions still fail to meet industrial-scale application requirements, which remains the primary challenge limiting the engineering popularization of MFC technology [49].

Studies on *Geobacter*-mediated MFCs not only broaden the application scenarios of functional microorganisms, extending their metabolic value from pollution remediation to energy conversion. Meanwhile, explorations of composite technologies such as constructed wetland−microbial fuel cells break the technical boundaries between environmental ecological engineering and bioelectrochemistry. They deliver an innovative technical paradigm for establishing low-energy-consumption and high-value-added pollution treatment systems, and are of great significance for advancing the low-carbon transition of environmental governance.

### 4.3. Role in the Biogeochemical Cycle

Biogeochemical cycling refers to the continuous migration, transformation, and circulation of key elements including carbon, nitrogen, phosphorus, and iron between biological communities and abiotic environments, which serves as the fundamental ecological mechanism for maintaining terrestrial and aquatic ecosystem stability and functional resilience. Deciphering microbially mediated biogeochemical processes is a core research topic for understanding the laws of material cycling in the Earth’s surface layer, while the mechanisms governing element cycling in anaerobic habitats have long remained insufficiently understood.

As a core functional genus of heterotrophic iron reduction, *Geobacter* participates extensively in the biogeochemical cycling of carbon, nitrogen, and iron via diverse metabolic pathways. Its outstanding extracellular electron transfer capability and metabolic endowments enable it to regulate elemental biogeochemical fluxes and sustain the structural and functional stability of subsurface ecosystems [50,51]. Furthermore, element cycling mediated by *Geobacter* can be coupled with heavy metal immobilization and organic pollutant degradation. Targeted regulation of biogeochemical processes constitutes the core logic of in situ bioremediation, and application-oriented demands have driven research expansion from basic theories to ecological regulation.

*Trichlorobacter* was first described as an independent genus in 2000, subsequently reclassified into the genus *Geobacter* in 2007, and then formally reinstated as a distinct genus following a comprehensive taxonomic reassessment in 2020. Despite repeated revisions to its nomenclature and taxonomic placement, this bacterium has consistently been recognized by the scientific community for its distinctive capacity to reduce and dechlorinate organic chlorinated compounds [52]. Chlorinated aliphatic hydrocarbons (CAHs), represented by 1,1,2-trichloroethane (1,1,2-TCA), are typical persistent, highly toxic, and carcinogenic groundwater contaminants that pose severe ecological risks. Anaerobic reductive dechlorination mediated by organohalide-respiring bacteria (OHRB) is recognized as a promising in situ bioremediation strategy. Microbial succession analyses consistently identify *Trichlorobacter* and *Dehalococcoides* as the dominant functional dechlorinating taxa in polluted groundwater systems [53]. Over the past two decades, relevant research has evolved from basic physiological characterization to multi-scenario applied exploration, forming a relatively complete and mature theoretical and technical system to support the sustainable development of this field.

The iron biogeochemical cycle is predominantly driven by microbial iron reduction mediated by *Geobacter* species, which occupies a central position in environmental iron elemental transformation. In constructed wetland ecosystems, conventional nitrogen removal relies on nitrification–denitrification and anaerobic ammonium oxidation pathways. The proliferation of anaerobic iron-reducing bacteria (primarily *Geobacter*) drives the reductive dissolution of iron oxides, triggering coupled multi-element biogeochemical cycling reactions [54]. As a tertiary wastewater treatment facility, constructed wetlands rely on microbial functional communities to realize pollutant purification. The colonization of aquatic macrophytes can significantly enrich core functional microorganisms involved in carbon and nitrogen cycling, including ammonia-oxidizing *Nitrosomonas* and iron-reducing *Geobacter* [55]. Notably, electroactive *Geobacter* strains capable of dissimilatory nitrate reduction to ammonium (DNRA) can maintain high biomass abundance via extracellular electron transfer to insoluble iron oxides. This iron-coupled nitrogen transformation mechanism provides a novel pathway for reactive nitrogen resource recovery from wastewater, offering new insights for constructing circular economic systems and revealing the interactive coupling relationships among carbon, iron, and nitrogen biogeochemical cycles [56]. As a model exoelectrogenic microorganism, *Geobacter sulfurreducens* is specialized in interacting with insoluble electron acceptors such as Fe(III) oxides, thus playing an irreplaceable role in both natural iron biogeochemical cycling and engineered microbial electrochemical systems [57].

Beyond its well-documented functions in iron and nitrogen cycling, *Geobacter* participates in multiple other biogeochemical processes. Emerging studies confirm that *Geobacter* species can regulate the emission of soil greenhouse gases including CO_2_, CH_4_, and N_2_O via redox-dependent metabolic transformations. Their metabolic activities are closely coupled with soil biogeochemical networks, thereby profoundly affecting regional greenhouse gas flux and carbon sequestration efficiency [58].

Thallium (Tl) is a highly toxic trace heavy metal with strong environmental mobility and bioaccumulation potential, posing serious threats to ecological security and human health. Large-scale mining and smelting activities of Tl-bearing sulfide minerals have caused widespread Tl contamination in river and soil environments, arousing extensive scientific concern. Microbial mediation dominates the geochemical transformation of Tl, and *Geobacter* species have been verified as key functional microorganisms regulating Tl redox speciation, solubility, and in situ immobilization in contaminated environments.

Paddy soil is a typical alternating oxic−anoxic redox environment with frequent elemental transformation reactions. *Geobacter* spp. are widely distributed in paddy soil systems and play a pivotal role in iron reduction and interfacial electron transfer processes. Recently, four novel iron-reducing bacterial strains affiliated with the family *Geobacteraceae* were isolated and taxonomically identified from typical agricultural paddy soils in Japan, further expanding the species diversity and functional research scope of soil *Geobacter* resources [59].

Research in this field not only improves the theoretical framework of synergistic multi-element cycling in anaerobic ecosystems, but also provides core theoretical support for natural attenuation assessment of contaminated sites and the development of in situ bioremediation technologies.

### 4.4. Application Research in Polluted Soil Remediation

With the advancement of global industrialization, environmental problems such as groundwater contamination by heavy metals, recalcitrant organochlorines and petroleum hydrocarbons have become increasingly severe. Conventional ex situ remediation technologies are limited by high costs, severe habitat disturbance and risks of secondary pollution. In situ bioremediation has therefore become a core development direction in pollution control owing to its eco-friendliness and thorough treatment effects. Owing to its dual functions in organic pollutant biodegradation and heavy metal reduction/immobilization, *Geobacter* exhibits unique advantages in the remediation of contaminated soil, groundwater, and sediment environments, serving as a core functional microbial resource for environmental bioremediation [60]. The practical demand for composite pollution remediation has further promoted research on coupling *Geobacter* with constructed wetlands, bioelectrochemical systems, and other technologies, continuously expanding its application scope.

Previous studies have constructed 16S rRNA gene and transcript clone libraries for three typical subsurface contaminated environments with indigenous dominant *Geobacter* communities, including uranium-contaminated aquifers undergoing in situ bioremediation, acetate-amended aquifer model systems, and oil-contaminated aquifers with aromatic hydrocarbon pollution. Relevant findings highlight the necessity of systematically exploring the behavioral characteristics and functional mechanisms of *Geobacter* in complex subsurface microbial communities, so as to achieve accurate simulation and efficient optimization of in situ bioremediation for metal and organic composite pollutants [61]. As two model iron-reducing bacterial genera, *Shewanella* and *Geobacter* are widely investigated for their bioremediation potential, iron reduction performance, and bioelectricity generation capacity. In freshwater lake sediments, the abundance and community diversity of iron-reducing bacteria are significantly correlated with eutrophication levels, demonstrating great application prospects of iron-reducing microorganisms in aquatic eutrophication mitigation and ecological restoration [62]. In oil-contaminated aquifers, the high expression of the benzoyl-CoA reductase subunit gene bamB in *Geobacter* species confirms their core functional status in the anaerobic degradation of aromatic hydrocarbon pollutants [63].

*Geobacter* species are critical microbial drivers for the in situ remediation of subsurface organic and heavy metal contaminants. In uranium-contaminated aquifers undergoing bioremediation, the proliferation of *Geobacter* populations significantly promotes the activity and abundance of indigenous *Geobacter*-associated bacteriophages. Future research needs to further explore the ecological functions of these bacteriophages and their regulatory effects on microbial community structure and remediation efficiency in actual contaminated sites [64].

In situ activation of indigenous metal-reducing microorganisms is a feasible and efficient strategy for uranium-contaminated groundwater remediation. Existing studies have verified the technical viability of this bioremediation approach; nevertheless, sustaining the long-term metabolic activity and population stability of *Geobacter* communities remains a key technical bottleneck restricting field remediation efficiency, requiring further optimization of field operational strategies [65].

*Geobacter* species can enzymatically reduce soluble toxic U(VI) to insoluble stable U(IV), while simultaneously transforming coexisting composite pollutants, making them the core functional microorganisms for targeted remediation of uranium-contaminated environments. The exogenous addition of organic electron donors to polluted groundwater for enhanced bioremediation will generate selective pressure on indigenous microbial communities. As a strain with efficient lactic acid metabolic capacity, *Geobacter sulfurreducens* has become the dominant model organism for exploring the physiological adaptation mechanisms of *Geobacter* under field remediation conditions [66].

*Geobacter metallireducens* also contributes substantially to environmental remediation practice. It can efficiently oxidize multiple aromatic organic pollutants in wastewater, playing a vital role in industrial wastewater purification [67]. Additionally, *G. metallireducens* possesses measurable decolorization and degradation capacity for various structurally diverse azo dye pollutants. Research on azo dye remediation by *Geobacter* spp. provides valuable theoretical support for revealing natural attenuation mechanisms and optimizing engineered remediation technologies for dye-contaminated water bodies and soils [68].

Research in this field bridges the translational chain between microbial physiological mechanism research and environmental engineering applications, offering low-carbon and eco-friendly solutions for pollution abatement in hard-to-intervene environments such as groundwater and deep soil.

Based on comprehensive analytical findings and the current status of field research, although existing studies on the genus *Geobacter* have established a relatively systematic research framework—encompassing extracellular electron transfer (EET) mechanisms, microbial fuel cell (MFC) development, functional interpretation of biogeochemical cycling, and bioremediation applications—several critical knowledge gaps persist. At the fundamental theoretical level, the synergistic, multi-factor regulatory mechanisms governing extracellular electron transfer remain incompletely elucidated. Interspecies electron transfer pathways—and the underlying interaction principles—between *Geobacter* and other functionally relevant microbial consortia within complex natural communities are still poorly defined. Moreover, the molecular network through which *Geobacter* mediates synergistic, multi-element biogeochemical cycling has yet to be fully characterized. In addition, the taxonomic evolutionary relationships and functional differentiation mechanisms between *Trichlorobacter* and *Geobacter* warrant rigorous, in-depth investigation. At the technological application level, microbial fuel cells continue to face significant bottlenecks: low energy conversion efficiency; high costs associated with core electrode materials; mass transfer limitations; and insufficient biofilm stability during scale-up to practical, large-scale deployment. Concurrently, *Geobacter*-mediated bioremediation technologies are constrained by limited strain tolerance to environmental stressors, inadequate capacity of individual strains to address complex, multi-contaminant scenarios, and a dearth of mature, standardized, and field-replicable engineering protocols. Furthermore, the majority of extant studies rely heavily on laboratory-based pure cultures and simplified simulated systems; correspondingly, research on in situ functional regulation of *Geobacter* in natural habitats—and on the dynamic interplay between environmental drivers and microbial community structure—remains comparatively scarce.

Future research in this field should prioritize targeted directions including precise elucidation and multi-factor directional regulation of the molecular mechanisms underlying extracellular electron transfer, synthetic biology-based targeted modification of strain functions and environmental adaptability, exploration of interspecies interaction rules within complex microbial consortia and construction of composite microbial remediation systems, engineering optimization and cost control of bioelectrochemical systems, as well as coupled regulatory mechanisms of multi-element cycling in natural habitats. These efforts will further smooth the translational pathway linking basic theories to engineering applications, enabling technologies related to *Geobacter* to deliver greater value in global environmental governance and new energy development.

## 5. Conclusions

This study systematically conducted a bibliometric and visual analysis of global research on *Geobacter* and *Trichlorobacter* spanning 2000–2026 based on 2223 peer-reviewed articles retrieved from the Web of Science Core Collection. Using CiteSpace and VOSviewer, this work comprehensively elucidated the 25-year developmental trajectory, thematic evolution, and interdisciplinary characteristics of this field, with a particular focus on four core research dimensions: extracellular electron transfer (EET), microbial fuel cells (MFCs), biogeochemical cycling, and environmental bioremediation. The findings provide data-driven insights for understanding the discipline’s evolutionary logic and guiding future fundamental and applied research.

Over the investigated period, *Geobacter*-related research experienced a two-stage evolutionary process, consisting of an initial foundational accumulation phase (2000–2010) and a subsequent rapid expansion phase (2011–2026), accompanied by continuous growth in global research outputs. Such robust development is primarily driven by the rising demand for sustainable environmental remediation, breakthrough advances in bioelectrochemical technologies, and deepening interdisciplinary integration of microbiology, electrochemistry, and environmental geoscience.

Collective publication and authorship data verify the formation of a stable, expanding academic community and a mature disciplinary publication system for *Geobacter* research. Keyword evolutionary analysis further reveals an evident research paradigm shift: research priorities have gradually transitioned from application-oriented technical exploration to in-depth mechanistic interpretation and systematic theoretical exploration. Future studies will focus on high-precision molecular mechanisms underlying *Geobacter* metabolic behaviors and EET processes, which will further boost technological innovation in environmental remediation, bioenergy conversion, and other interdisciplinary applications.

Keyword evolutionary analysis indicates that the research focus of this field has gradually shifted from applied technological exploration to fundamental mechanism interpretation and systematic theoretical construction. Future research on *Geobacter* metabolic pathways and extracellular electron transfer mechanisms will further develop toward molecular refinement, mechanistic precision, and systematic integration. These in-depth mechanistic investigations will effectively promote technological innovation and industrial upgrading in environmental remediation, bioelectrochemical energy conversion, and other interdisciplinary application fields.

Meanwhile, this study has several obvious limitations. First, all data were retrieved only from the Web of Science database, without including publications from other databases, which results in certain language bias and incomplete data coverage. Second, citation lag is inevitable: citation records of newly published papers have not been fully accumulated, potentially impairing the integrity of analyses related to highly cited literature. Follow-up research will establish a more comprehensive and systematic bibliometric framework to deepen the identification of research hotspots and developmental trends, facilitate higher-quality advances in this field, and fully unlock the core value of versatile electroactive strains within the genus *Geobacter*.

## 6. Perspective

(1) The extracellular electron transfer (EET) efficiency of *Geobacter* is highly susceptible to complex environmental variables, including ambient pH and temperature. However, quantitative models that systematically characterize the synergistic and antagonistic interactions among multiple physicochemical factors remain limited, restricting the precise modulation of EET performance under field conditions. In natural ecosystems, *Geobacter* rarely exists as pure cultures but coexists within complex multispecies microbial consortia. The interspecific electron transfer mechanisms and synergistic interactions between *Geobacter* and indigenous microbes remain poorly resolved, constituting a key bottleneck for improving the practical efficacy of in situ bioremediation and anaerobic digestion systems. Although various electrode modifications and nanomaterial interventions have been developed to amplify *Geobacter*-mediated EET, the performance enhancement is still modest and insufficient to meet the energy efficiency requirements of large-scale engineering deployment. Moreover, persistent engineering challenges, including interfacial mass transfer limitations and biofilm detachment during reactor scale-up, have not been effectively addressed. Future research should integrate molecular microbiology, electrochemistry, materials science, and systems biology to decode the core molecular regulatory mechanisms of EET and establish comprehensive multi-factor regulatory and predictive frameworks.

(2) *Geobacter*-driven microbial fuel cells (MFCs) perfectly align with the global pursuit of sustainable wastewater treatment and low-carbon energy production under the dual-carbon strategic framework. Nevertheless, their large-scale practical translation is hindered by several critical technical and economic bottlenecks, including low overall energy conversion efficiency, unmitigated electron loss during EET processes, high fabrication and material costs, insufficient long-term operational stability, and poor strain tolerance to fluctuating and inhibitory wastewater environments. To bridge the lab-to-field translational gap, future research requires coordinated advances in fundamental mechanism research and applied engineering optimization. Priority should be given to the rational design and scalable fabrication of low-cost, high-performance electrode materials to reduce system capital costs. Furthermore, strain engineering and microbial community modulation strategies should be adopted to enhance the environmental adaptability, functional robustness, and stress resistance of *Geobacter* in complex dynamic environments, thereby accelerating the transformation of MFC technology from laboratory proof-of-concept to practical low-carbon wastewater treatment solutions.

(3) While the pivotal contributions of *Geobacter* to multi-element biogeochemical cycling have been widely acknowledged, the underlying molecular regulatory mechanisms remain incompletely understood. In particular, how *Geobacter* orchestrates coupled elemental transformation processes via EET and integrated metabolic networks requires systematic clarification. Additionally, the interspecific interactions between *Geobacter* and co-occurring microbial communities in natural habitats, as well as their collective impacts on the efficiency and stability of biogeochemical cycling, remain elusive. Future studies should focus on dissecting the fine-scale mechanisms of *Geobacter*-mediated elemental cycling, so as to establish a more systematic theoretical framework for understanding ecosystem material circulation, guiding in situ environmental regulation, and supporting ecological restoration and stability maintenance.

(4) Although *Geobacter*-based bioremediation has achieved prominent progress in laboratory- and pilot-scale trials, its large-scale field application still faces multiple unresolved challenges. Wild-type *Geobacter* strains generally exhibit limited environmental adaptability and stress tolerance, leading to compromised viability and pollutant degradation performance under extreme conditions such as high pollutant loads and fluctuating pH. Furthermore, the remediation spectrum of single strains is inherently restricted, making them inadequate for addressing complex multi-pollutant contamination scenarios. More importantly, standardized, scalable, and field-verified remediation technologies and systematic engineering protocols are still lacking, hindering the industrial popularization of *Geobacter* bioremediation. To break through these translational limitations, future research should focus on three strategic directions: first, applying synthetic biology and precise genome editing technologies to construct engineered strains with enhanced stress resistance and optimized EET efficiency; second, designing and constructing functionally synergistic multispecies microbial consortia to broaden pollutant degradation spectra and improve remediation stability under mixed contamination; and third, developing and validating site-specific, replicable remediation frameworks to promote the standardized deployment and practical application of *Geobacter*-based environmental remediation technologies.

## Figures and Tables

**Table 1:** Top 10 authors, top 10 cited authors, and top 10 journals.

**Rank**	**Author**	**Institution**	**Publications**
1	Lovley Derek R	University of Massachusetts Amherst (USA)	177
2	Nevin Kelly P	University of Massachusetts Amherst (USA)	61
3	Salgueiro Carlos A	Universidade Nova de Lisboa (Portugal)	40
4	Wang Xin	Nankai University (China)	32
5	Li Nan	Tianjin University (China)	32
6	Holmes Dawn E	University of Massachusetts Amherst (USA)	32
7	Feng Yujie	Harbin Institute of Technology (China)	27
8	Dantas Joana M	Universidade Nova de Lisboa (Portugal)	22
9	Logan Bruce E	Pennsylvania State University (USA)	22
10	Malvankar Nikhil S	Yale University (USA)	20
**Rank**	**Author**	**Total Citation Frequency**	**Country**
1	Lovley Derek R	1149	USA
2	Logan BE	665	USA
3	Bond DR	479	USA
4	Holmes DE	468	USA
5	Reguera G	465	USA
6	Nevin KP	420	USA
7	Malvankar NS	326	USA
8	Rotaru AE	295	UK
9	Rabaey K	275	Belgium
10	Marsili E	236	China
**Rank**	**Source**	**Documents**	**Citations**
1	*Bioresource Technology*	142	8528
2	*Water Research*	93	5411
3	*Science of The Total Environment*	92	2872
4	*Applied and Environmental Microbiology*	80	10,967
5	*Environmental Science & Technology*	72	6030
6	*Chemical Engineering Journal*	70	2724
7	*Frontiers in Microbiology*	69	2599
8	*Journal of Hazardous Materials*	65	2599
9	*Chemosphere*	50	1469
10	*Bioelectrochemistry*	49	1506

**Table 2:** Top 10 institutions in *Geobacter*-related research (2000–2026).

Rank	Organization	Country	Publications
1	University of Massachusetts	USA	200
2	Chinese Academy of Sciences	China	172
3	Harbin Institute of Technology	China	134
4	University of Chinese Academy of Sciences	China	57
5	Pacific Northwest National Laboratory	USA	53
6	NOVA University Lisbon	Portugal	53
7	Tianjin University	China	50
8	Nankai University	China	50
9	The Pennsylvania State University	USA	49
10	Tsinghua University	China	46

## Data Availability

The original contributions presented in this study are included in the article. Further inquiries can be directed to the corresponding author.

## Supplementary Materials

The following supporting information can be downloaded at: https://www.mdpi.com/article/10.3390/microorganisms14081678/s1; Table S1: PRISMA 2020 Checklist.
